# Development of a V_H_H-Based Erythropoietin Quantification Assay

**DOI:** 10.1007/s12033-015-9860-7

**Published:** 2015-03-13

**Authors:** Stefan Kol, Thomas Beuchert Kallehauge, Simon Adema, Pim Hermans

**Affiliations:** 1 The Novo Nordisk Foundation Center for Biosustainability, Technical University of Denmark, Kogle Allé 6, 2970 Hørsholm, Denmark; 2 BAC BV, Life Technologies, Huizerstraatweg 28, 1411 GP Naarden, The Netherlands

**Keywords:** Chinese hamster ovary (CHO) cells, Erythropoietin, EPO, Biolayer interferometry, Quantification assay, VHH

## Abstract

Erythropoietin (EPO) quantification during cell line selection and bioreactor cultivation has traditionally been performed with ELISA or HPLC. As these techniques suffer from several drawbacks, we developed a novel EPO quantification assay. A camelid single-domain antibody fragment directed against human EPO was evaluated as a capturing antibody in a label-free biolayer interferometry-based quantification assay. Human recombinant EPO can be specifically detected in Chinese hamster ovary cell supernatants in a sensitive and pH-dependent manner. This method enables rapid and robust quantification of EPO in a high-throughput setting.

## Introduction

Erythropoietin (EPO) is a 34 kDa glycoprotein hormone produced in the adult kidney and in the fetal liver. It controls erythropoiesis by stimulating maturation of erythroid precursor cells in the bone marrow through interaction with the EPO receptor expressed on its surface, resulting in increased numbers of red blood cells [1]. EPO comprises a 165 amino acid protein with 40 % of its mature molecular weight accounted for by carbohydrates. The structure contains three N-glycans located on asparagine residues at positions 24, 38, and 83 and one mucin-type O-glycan located on the serine 126 residue [2, 3]. Due to this complexity, EPO intended for therapeutic use is produced in mammalian host cells, mainly in Chinese hamster ovary (CHO) cells. Recombinant human EPO is now available in sufficient amounts for clinical use in the treatment of anemia that results from chronic renal failure [4].

Accurate quantification techniques are critical for the selection of high producing clones during stable cell line development and optimization of bioreactor titers in production. Traditional measurement methods include ELISA, HPLC or densitometric analysis of Western blots. Despite the widespread adoption of ELISA in industry, there are several drawbacks to this method, e.g., high reagent cost, long total assay time, and extensive ‘hands-on’ time resulting in a high variability in results and susceptibility to human error. Due to its high degree of automation, robustness, and reproducibility liquid chromatography is the workhorse of the biopharmaceutical laboratory, but throughput is limited to at most 4–12 samples per hour. Quantitative Western blotting is time consuming and challenging to perform properly, and it has therefore not been adopted on a large scale. Faster times-to-results, better consistency, and less human intervention are the prerequisites for the development of high-throughput laboratory quantification methods.

Bio-layer interferometry (BLI) is an emerging technology that offers label-free quantification of antibodies and other proteins [5, 6]. Conventional BLI quantification assays use an immobilized full-length antibody to achieve the specificity to detect non-antibody proteins. Sensitivity can be improved by employing a two-step ELISA-like sandwich assay. To increase sensitivity even further a three-step assay can be employed that uses a secondary HRP-linked antibody to amplify detection signals. We sought to develop a direct, one-step binding assay which simplifies data interpretation and reduces assay time. For this purpose, a camelid single-domain antibody fragment directed against human EPO (anti-EPO V_H_H) was evaluated as a capturing antibody. V_H_H fragments are the smallest intact antigen binding domains derived from heavy chain-only antibodies of camelids [7]. They are very stable, highly soluble, and can easily be produced recombinantly [7, 8]. These binding domains have been successfully applied as affinity ligands in chromatography processes covering a variety of biotherapeutics [9–12], but can also serve as target-specific capture reagents for label-free technologies like BLI [13]. Since in BLI the shift in wavelength, Δλ, is a direct measure of the change in optical thickness close to the surface of a biosensor tip, the use of a V_H_H-based affinity ligand for capture that is only one-tenth in size of a conventional 150 kDa antibody should lead to a higher sensitivity in a BLI-based quantification assay.

Here, we describe the development of a sensitive and robust EPO quantification assay using BLI. Our anti-EPO V_H_H pre-coated immunosensors specifically bind EPO in a pH-dependent fashion. The detection range using 200 s incubation was 0.31–20 μg/ml spiked EPO. Although initially a large amount of unspecific binding in CHO cell supernatants was detected, we were able to strongly reduce these signals by increasing the ionic strength of the solution. Immunosensors can be regenerated using high-pH conditions and reused for up to 10 rounds of quantification. The results from an absolute EPO quantification correspond well to those obtained using a commercially available EPO ELISA kit.

## Materials and Methods

### Cloning of Expression Constructs

Human EPO sequence was retrieved from Uniprot P01588 (EPO_HUMAN), PCR amplified, and cloned into pcDNA3.1(+) vector using NheI and EcoRI sites. A codon-optimized gene for EPO (coEPO) was ordered from GeneArt and cloned in the same manner. The synthetic sequences of Rituximab heavy and light chain were ordered codon optimized for CHO from GeneArt. Sequences were based on protein sequences from the DrugBank database under accession number DB00073. An N-terminal signal peptide (MGWSCIILFLVATATGVHS) was added to both protein sequences. Rituximab heavy chain and light chain were cloned into the dual expression vector pBudCE4.1 (Life Technologies, Carlsbad, CA, USA). Empty vector control was pcDNA3.1(+). Rituximab heavy chain was PCR amplified and cloned using SalI and BamHI, and Rituximab light chain was PCR amplified and cloned using NotI and XhoI.

### Cell Culture and Transient Transfection

CHO-S suspension cells (Life Technologies) were grown in CD-CHO medium (Life Technologies) supplemented with 8 mM glutamine (Gibco/Life Technologies). Cells were maintained in flat-bottomed 125 mL Erlenmeyer flasks (Corning # 734-1885) and incubated at 37 °C, 5 % CO_2_ at 120 rpm. Passage cells culture was supplied with 1 μL/mL anti-clumping agent (Life Technologies). Anti-clumping agent was removed one day prior to transient transfection. 10^6^ cells were transfected in 10 mL CD-CHO supplemented with 8 mM Glutamine. Cells were transfected with plasmid DNA at a concentration of 1.25 μg/10^6^ cells using FreeStyle™ MAX reagent (Life Technologies) according to the manufacturer’s protocol. Anti-clumping agent was added to transfected cells 24 h post-transfection at a concentration of 1.5 μL/mL. After 96 h, supernatants were harvested by pelleting cells by centrifugation (10 min, 1000 rcf) and 0.22-μm filtration. Conditioned medium was generated by propagating CHO-S suspension cells for 96 h and harvesting the supernatant. A stable cell line of CHO-S expressing the codon-optimized gene for EPO, a stable cell line of CHO-GS expressing His-tagged EPO, and several transient transfected cell lines (CHO-S expressing EPO and CHO-S expressing Rituximab) were used in the ELISA comparison experiment. Where indicated, supernatants were harvested on day 0, 1, 2, and 3 after transfection or expansion.

### Assay Conditions

Biolayer interferometry was performed using an Octet RED96 (Pall, Menlo Park, CA, USA). Streptavidin kinetic grade biosensors (Fortebio 18-5021, Pall) were hydrated in PBS, functionalized with the anti-EPO V_H_H biotin conjugate (Life Technologies, cat.no: 7103372100) or a biotinylated anti-EPO IgG (AB-286-NA, R&D systems, Minneapolis, MN, USA) at 5 μg/ml in PBS, and blocked in PBS containing 1 μg/ml biocytin (120, 600, and 300 s, respectively). The anti-EPO IgG was biotinylated using the EZ-Link biotinylation kit (Thermo Scientific, Rockford, IL, USA) with a ratio of biotin:antibody of 1. EPO dilution series were prepared using commercially bought EPO (Genscript, Piscataway, NJ, USA) and our in-house purified EPO at 20, 10, 5, 2.5, 1.25, 0.625, 0.3125 μg/ml in assay buffer (20 mM citric acid, 0.1 % BSA (w/v), 0.02 % tween-20, pH 4.0). After equilibration in assay buffer (120 s), EPO dilution series were measured for 200 s. The pH-dependent binding profile was tested with an EPO dilution of 10 μg/ml in 20 mM citric acid, 0.1 % BSA (w/v), 0.02 % tween-20, pH 4.0, 4.6, 5.2, 5.8, 6.4, 7.0, 7.6. Immunosensors were equilibrated in the same buffers without added EPO. Salt-dependent background reduction was performed using EPO and control CHO supernatants adjusted to sample diluent conditions with a 10 times concentrated buffer containing 200 mM citric acid, 1 % BSA (w/v), 0.2 % tween-20, pH 4.0. NaCl was added from a 5-M stock solution. Regeneration buffer scouting was performed at an EPO concentration of 20 μg/ml in the following solutions: 100 mM glycine pH 2.0, 100 mM phosphoric acid, 10 mM HCl, 10 mM Na_2_HPO_4_ pH 12, 5 M NaCl, 0.01 % Sodium dodecyl sulfate (SDS), 10 mM NaOH and 10 mM glycine pH 1.7. Regeneration was performed using three cycles of 5 s incubation in regeneration solution and 5 s in neutralization solution (PBS). Association was performed for 200 s with a shaking speed of 1000 rpm at 30 °C. All assays were performed in 96-well black microplates (Greiner Bio-One 655209). Octet System Data Analysis 7.1 software was used to calculate binding rates and absolute EPO concentrations (standard curve equation: Dose response 4PL, binding rate equation: R equilibrium). Storage incubation buffer was PBS containing 15 % sucrose.

### Other Methods

His-tagged EPO was purified from the supernatant of a stable hisEPO-expressing CHO-S cell line using a HisTrap Excel affinity column and HiLoad 16/600 superdex 200 pg gel filtration column (GE Healthcare, Piscataway, NJ, USA), both according to the manufacturer’s instructions. The Human EPO ELISA Kit (Stem cell technologies, Vancouver, BC, Canada) was performed according to the manufacturer’s instructions. Western blotting was performed using a standard protocol except that a streptavidin-peroxidase conjugate (Thermo Scientific) was used to visualize the V_H_H biotin conjugates. The V_H_H biotin conjugates were diluted to a working concentration of 5 μg/ml. Rituximab was detected using a rabbit anti-human IgG HRP-conjugate (Fisher Scientific, Waltham, MA, USA). Anti-EPO monoclonal IgG (ab19485, Abcam, Cambridge, UK) was diluted 1:1000.

## Results

### The V_H_H Ligand Specifically Binds EPO and Can Be Applied as an Immunosensor

**Fig. 1:**
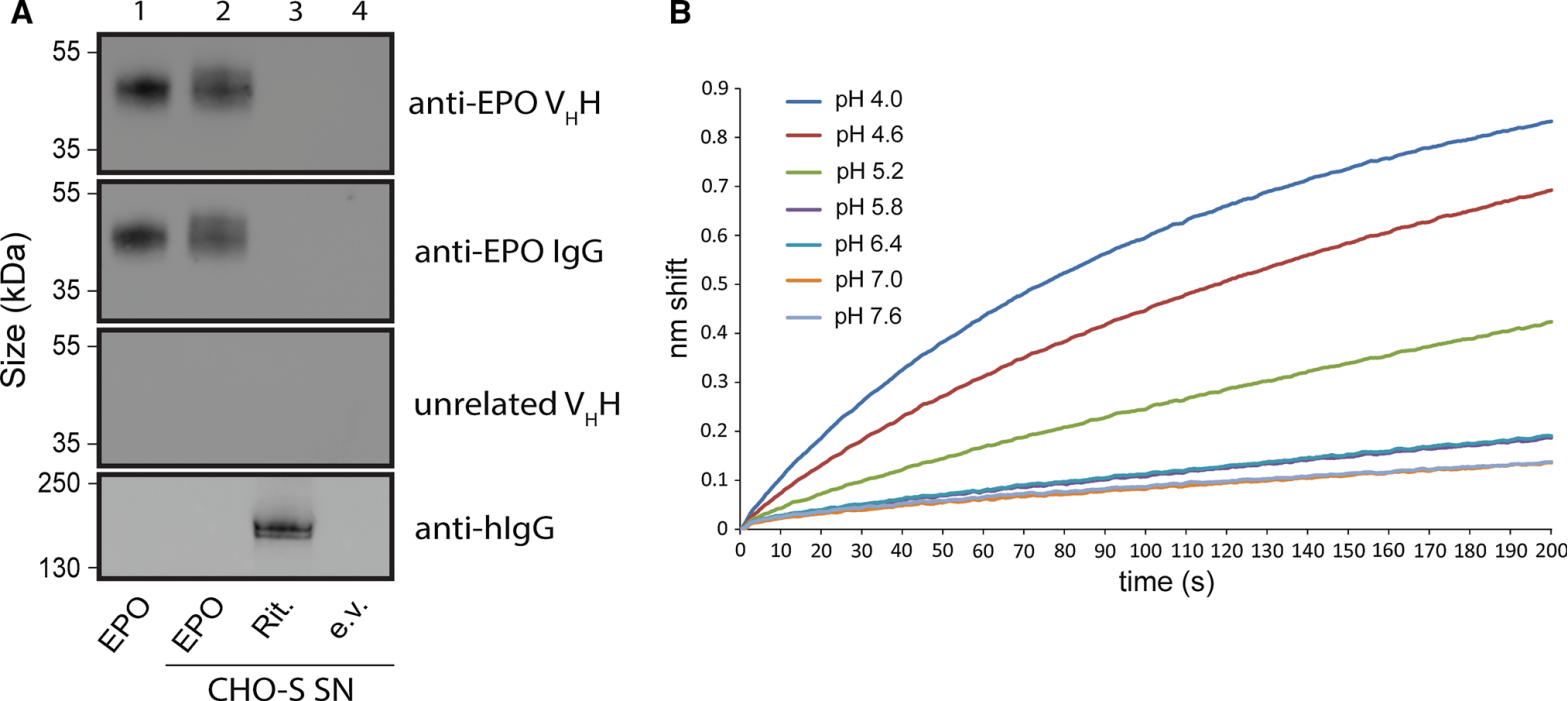
Anti-EPO V_H_H specificity and pH dependence. **a** Western blot of 0.1 μg purchased EPO (*lane 1*) and 10 μl of CHO cell supernatant transfected with a vector containing the human EPO gene (*lane 2*), a vector containing the Rituximab heavy and light chains (*lane 3*), and an empty vector (*lane 4*). Blots were developed using anti-EPO V_H_H (*top panel*), anti-EPO IgG (*second panel*), a V_H_H raised against an unrelated glycoprotein (*third panel*), and an anti-human IgG-HRP conjugate (*lower panel*). **b** Sensorgrams showing immobilized anti-EPO V_H_H binding of in-house purified EPO (10 μg/ml) at the indicated pH values

To verify the specificity of the anti-EPO V_H_H, Western blotting analysis was performed on commercially available purified EPO and on supernatants of CHO cells expressing either Rituximab or EPO. A supernatant from cells transfected with an empty vector served as a negative control. Biotinylated anti-EPO V_H_H was used as the primary immuno-detection step, while a streptavidin-HRP fusion was used for secondary detection. Due to its extensive glycosylation, purified EPO migrates as a smear of around 45 kDa (Fig. 1a, anti-EPO V_H_H, lane 1). In the supernatants from CHO cells expressing EPO a similar smear was detected, while no background bands or smears could be seen (Fig. 1a, anti-EPO V_H_H, lane 2). Rituximab and empty vector control supernatants displayed no interaction with the anti-EPO V_H_H (Fig. 1a, anti-EPO V_H_H, lane 3 and 4). A conventional anti-EPO IgG served as a positive control and was used to confirm the presence of EPO (Fig. 1a, anti-EPO IgG, lane 1 and 2). We also performed detection with a biotinylated V_H_H raised against an unrelated glycoprotein in the same manner as the anti-EPO V_H_H. We found that it does not cross-react with EPO or any other proteins found in CHO-S supernatants (Fig. 1a, unrelated V_H_H). The presence of Rituximab was confirmed using an anti-human IgG (Fig. 1a, anti-hIgG). These results show that the anti-EPO V_H_H is highly specific for EPO.

To construct an immunosensor, the anti-EPO V_H_H biotin conjugate was immobilized onto streptavidin (SA) biosensors. Functionalization of the tips with the V_H_H biotin conjugate reproducibly resulted in a shift of 3.5 nm (data not shown). Previous experiments where the anti-EPO V_H_H was used as an affinity purification resin had indicated that its binding behavior is severely affected by changes in pH (Pim Hermans, unpublished observations). To define the optimal pH-range for EPO detection, we performed a pH scouting experiment using in-house purified EPO spiked into CHO supernatants. After equilibration of previously prepared immunosensors in EPO-free pH-adjusted buffer, we measured EPO for 200 s at pH 4.0, 4.6, 5.2, 5.8, 6.4, 7.0, and 7.6. Results show that the highest nm shift within this pH range was detected at pH 4.0 (Fig. 1b). At higher pH values the detected nm shift was severely reduced, while around neutral pH almost no binding was observed. We conclude that EPO binding under these experimental conditions was also strongly influenced by the pH. Assay conditions were set to pH 4.0 in all subsequent experiments.

**Fig. 2:**
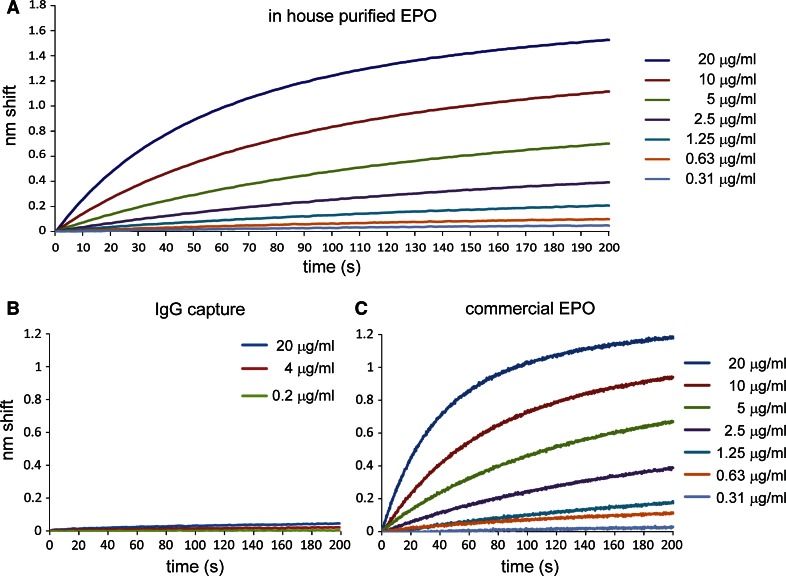
Immunosensor sensitivity. Representative sensorgrams showing immobilized anti-EPO V_H_H binding of in-house purified EPO (**a**), showing immobilized anti-EPO IgG binding of in-house purified EPO (**b**), and showing immobilized anti-EPO V_H_H binding of commercial EPO (**c**). EPO was present at the indicated concentrations

To assess the sensitivity of the anti-EPO immunosensors, we prepared a dilution series of in-house purified his-tagged EPO. Incubation for 200 s resulted in a maximum shift of about 1.55 nm (Fig. 2a). The maximum shift decreased with decreasing EPO concentrations, but even at the lowest concentration of EPO used (0.31 μg/ml), we still detected a shift of about 0.05 nm. When we employed a conventional IgG as a capture molecule, we only observed a maximum shift of 0.05 nm at 20 μg/ml EPO (Fig. 2b), while this signal rapidly decreased at lower concentrations of EPO. We also compared the results from in-house purified EPO to a dilution series of commercially available EPO (Fig. 2c). The maximum shift of about 1.2 nm was lower than the shift detected with in-house purified EPO. This decrease in signal was also observed at the lowest concentration of EPO, where a shift of about 0.02 nm was observed. Besides giving a higher signal, the curves generated for our in-house purified EPO also appear to be more smoothed than the ones generated for commercial EPO. The detection range of the anti-EPO V_H_H immunosensors using 200 s incubation was 0.31–20 μg/ml spiked EPO. These results show that our immunosensors can detect a range of EPO with short incubation times.

### Unspecific Binding in CHO Supernatants Reduced by Increasing Ion Strength

**Fig. 3:**
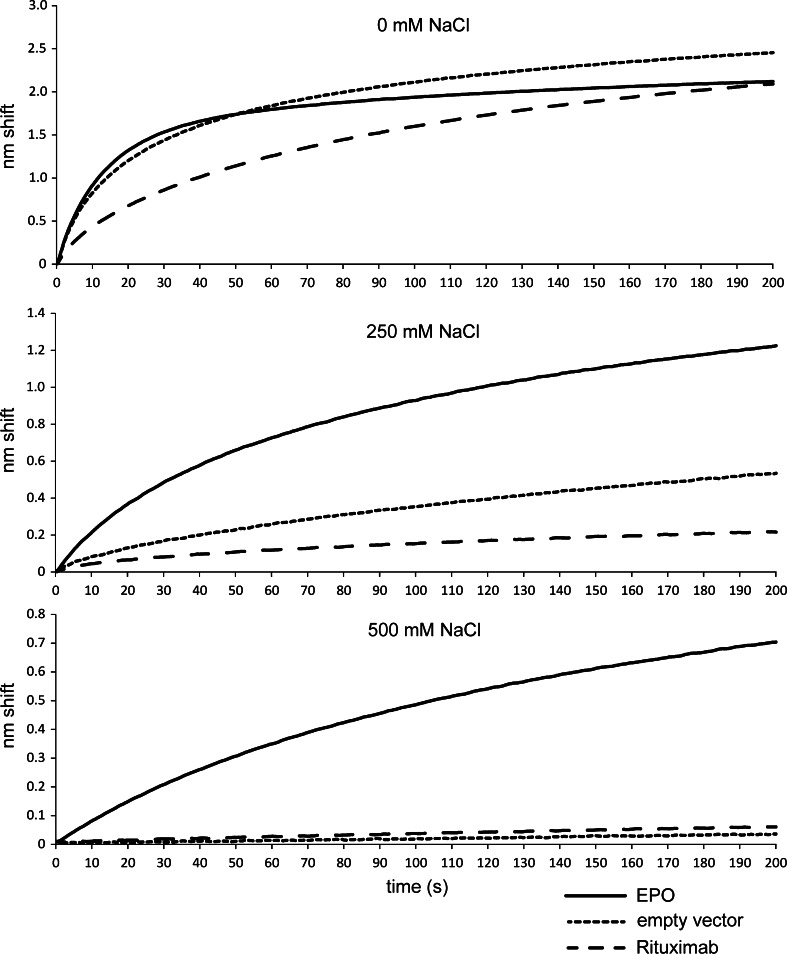
Background reduction by increased ionic strength. Sensorgrams generated using supernatants of CHO cells transfected with an empty vector (*dotted line*), a vector containing the human EPO gene (*solid line*), or a vector containing the Rituximab heavy and light chains (*dashed line*). NaCl was added at the indicated concentrations

Next we assessed if EPO could be detected in supernatants from transient EPO expressions in CHO cells. Previous prepared immunosensors were used to measure in EPO, Rituximab, and empty vector supernatants. When measuring empty vector and Rituximab supernatants, we detected a large shift (~2.5 nm) that was comparable to the shift obtained for an EPO containing supernatant (Fig. 3, top panel). As these shifts were much larger than the shift detected for the highest concentration of EPO in buffer (Fig. 2) and there was little difference between control and EPO supernatants, we speculated that there was a high degree of unspecific binding within the supernatants. Upon raising the ionic strength of the CHO supernatants by the addition of 250 mM NaCl, the total nm shift was reduced strongly for all samples, while an EPO-specific shift of about 1.2 nm was detected (Fig. 3, middle panel). The unspecific binding of empty vector and Rituximab supernatants was reduced almost completely by increasing the ionic strength even further (Fig. 3, lower panel). The EPO-specific shift was also further reduced to about 0.7 nm. When the ionic strength was raised even more, a high unspecific binding during equilibration and a negative nm shift in supernatants was observed (data not shown). These results show that addition of 500 mM NaCl was optimal for the detection of EPO in CHO cell supernatants as it minimizes unspecific signals, while only the EPO-specific signal remains.

### Regeneration and Storage of Functionalized Biosensor Tips

**Fig. 4:**
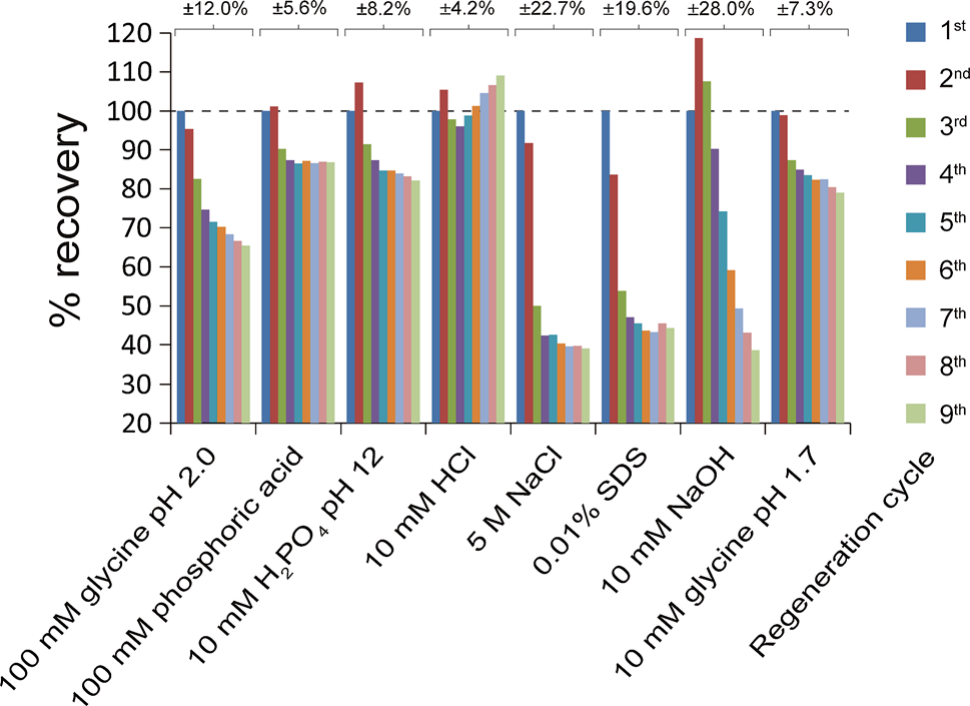
Biosensor regeneration. Wild-type CHO-S supernatants spiked with in-house purified EPO were measured nine times after an initial round of pre-conditioning. The first round of binding was set at 100 % and subsequent rounds of binding were expressed as a percentage thereof. Regeneration was performed in the indicated solutions. Standard deviation is indicated above the columns

Regeneration allows for reuse of immunosensors for subsequent rounds of analysis and a reduction of costs. By disrupting protein–protein interactions between the immobilized V_H_H and the analyte, the functionalized biosensor can directly be reused for the next round of analysis or stored for later use. Typically interactions can be efficiently disrupted using low pH, high pH, high salt concentrations or detergents. We decided to test the following regeneration buffers: 100 mM glycine pH 2.0, 100 mM phosphoric acid, 10 mM HCl, 10 mM Na_2_HPO_4_ pH 12, 5 M NaCl, 0.01 % SDS, 10 mM NaOH and 10 mM glycine pH 1.7. After pre-conditioning through a single round of equilibration and regeneration, the anti-EPO immunosensors were used to measure a known concentration of EPO spiked into CHO supernatant (Fig. 4). The nm shift of the first EPO measurement was set as 100 percent and subsequent measurements were expressed as a percentage thereof. The regeneration conditions that show close to 100 percent recovery after repeated regenerations and show a low standard deviation are good regeneration candidates. A rapid decrease in binding was detected when using NaCl and SDS as regeneration reagents, showing that the binding properties of the anti-EPO V_H_H functionalized biosensors are severely disrupted after incubation under these conditions. All the low-pH conditions had a similar regeneration profile, where the binding properties were still intact after the first incubation, but decrease to 65–80 % recovery after several rounds of regeneration (100 mM glycine pH 2.0, 10 mM HCl and 10 mM glycine pH 1.7) or remain constant at about 85 % recovery (100 mM phosphoric acid). Regeneration in 10 mM NaOH leads to an increase in signal after the first two binding events, but recovery rapidly decreased thereafter to about 40 %. The other high pH condition tested (10 mM Na_2_HPO_4_ pH 12) showed some fluctuation in binding recovery. After an initial increase, the recovery decreased to about 96 % in the 4th regeneration. After that, the recovery steadily increased to 109 % in the 9th regeneration. Despite these fluctuations, the recovery percentage never differs more than 9 % from the value obtained in the first binding. The standard deviation was also the lowest under the conditions tested. Sodium phosphate adjusted to a high pH was therefore a suitable regeneration agent and it was used in subsequent experiments. Functionalized tips are used in the laboratory on a regular basis and have been stored for up to 2 weeks after a short incubation in storage buffer and subsequent dehydration without loss of sensitivity.

### Absolute Quantification Values of EPO Correspond Well with Values Obtained by ELISA

**Fig. 5:**
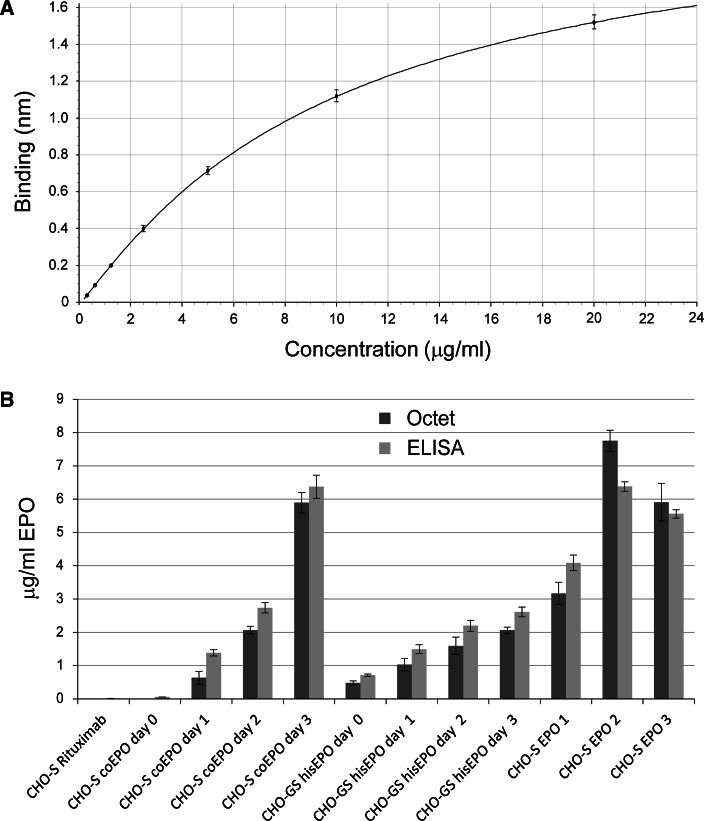
Comparison of Octet and ELISA absolute quantification. **a** Standard curve generated from dilution series of in-house purified EPO of known concentration using end-point values. Samples were run in triplicate. **b** Absolute EPO concentrations were determined of supernatants harvested from a stable cell line of CHO-S expressing the codon-optimized gene for EPO (coEPO), a stable cell line of CHO-GS expressing His-tagged EPO, and several transient transfected cell lines (3× CHO-S expressing EPO and CHO-S expressing Rituximab). Where indicated, supernatants were harvested on day 0, 1, 2, and 3. Empty vector supernatants were used as blank and samples were analyzed in triplicate in both assays

The results obtained by the Octet quantification assay were compared to a commercially available ELISA sandwich assay. Analyses were performed on supernatants from a panel of stable and transient transfected cell lines harvested at different time points. Absolute concentrations of EPO were calculated by comparison with a calibration curve generated from three separate dilution series of in-house purified EPO (Fig. 5a) or from the EPO dilution series included in the ELISA kit. No or very little EPO was detected in the Rituximab control supernatant (Fig. 5b) and these values were comparable to the values obtained from the empty vector blank supernatant. Although minor differences can be observed, native and tagged EPO concentrations in stable and transiently transfected cell lines correspond well between the two assays (Fig. 5b). An increase in titer can be observed for the EPO samples taken at different time points. The three EPO supernatants harvested from a transient transfected CHO-S cell lines differ from each other in EPO concentration. Furthermore, sample CHO-S EPO 1 and 2 also show the largest difference in concentration between the two assays. In general, the absolute concentrations acquired by our novel method agree very well with the values obtained by ELISA.

## Discussion

We present here a novel method to quantify secreted EPO produced in CHO cell lines. To the best of our knowledge, no prior studies describe a BLI-based quantification assay for non-antibody proteins. A method has been described for the quantification of Recombinant Human Factor IX in bioreactor harvests using a conventional antibody [14], but the method is only accessible through the website of Fortebio, the company that produces the Octet BLI system. In this work, we employ a biotinylated V_H_H to increase sensitivity and facilitate data interpretation. Application of a V_H_H as an immunosensor offers increased sensitivity over a conventional IgG in the one-step binding assay described here.

Concentration or clean-up of CHO cell supernatants is not needed, but samples need to be adjusted to the right pH and ionic strength. In addition, samples may need to be diluted to an EPO concentration below 20 μg/ml. In these experiments the lower limit of detection was 0.31 μg/ml of spiked EPO. Using a full 96-well microplate including two columns for regeneration and neutralization, we were able to measure 80 samples in 45 min using only 8 biosensors. As the V_H_H can be recovered and stored after construction of the immunosensor, the total cost for measuring a full plate is about €40. The method offers a very rapid and facile way to determine EPO titer as compared to ELISA or HPLC analysis. Because of its short setup and analysis time, it is especially well-suited for bioreactor sampling or for the selection of high producing clonal cell lines.

As shown by the comparison with ELISA, addition of a His-tag to the native EPO protein sequence, expression in different cell lines and stable versus transient expression does not alter binding to the immunosensor and thus does not influence absolute quantification. The differences we observed between in-house purified EPO and commercial EPO—lower nm shift and a more ‘noisy’ signal—are not readily explainable, but could be caused by deviations from the concentration declared by its manufacturer, by loss of sensitivity due to buffer additives like phosphates, or by differences in post-translational modifications. As the absolute quantification values of EPO calculated from a dilution series of in-house purified EPO agree well with the values obtained by ELISA, these preparations are used as calibration in our laboratory.

It is important to recognize that the extensive glycosylation on EPO may influence the results obtained by BLI quantification. A proteinaceous epitope is suggested by the observations that anti-EPO V_H_H is able to cross-react with non-glycosylated EPO derived from *Escherichia coli* by Western blotting and by its binding affinity of ~2 nM (Pim Hermans, unpublished observations). However, as its molecular mass consists of more than 40 % carbohydrates, deglycosylation will influence optical thickness close to the surface of a biosensor tip and thus the amount of shift which can be achieved. Deglycosylation will therefore lead to an underestimation of the actual concentration. If an introduced glycosylation site or a more extensive glycosylation pattern affects the epitope recognized by the anti-EPO V_H_H, binding may be completely abolished. The method described here is therefore not well suited for studies where a drastic change in glycosylation pattern is expected.

The experiments described here were performed with SA kinetic grade biosensors. Sensitivity could possibly be improved using super streptavidin (SSA) biosensors or the recently introduced high precision streptavidin (SAX) biosensors. The SAX biosensors have been specifically developed by Fortebio to perform high-precision quantification, but were not available yet during the design of this study. It remains to be seen whether these biosensors can improve the sensitivity of the assay described in this work. The throughput of this assay can also be improved using the Octet RED384 or HTX BLI platforms. The method is a promising candidate for further establishment and cross-validation against current reference methods. Moreover, the concept presented here can easily be modified using V_H_H ligands directed towards other biotherapeutic (glyco) proteins, using other types of small binding ligands like DARPins [15] or adapted for other label-free detection systems like those based on surface plasmon resonance (SPR).

## Abbreviations

HRP: Horseradish peroxidase
ELISA: Enzyme-linked immunosorbent assay
HPLC: High-performance liquid chromatography
